# Cardiovascular abnormalities in children with Turner syndrome: a 15-year retrospective study and analysis of warning signs

**DOI:** 10.3389/fped.2025.1576434

**Published:** 2025-08-11

**Authors:** Hongliang Jia, Ye Li, Panwang Huang, Beilei Zeng, Yuan Zhou, Zhuangjian Xu, Yaping Ma

**Affiliations:** ^1^ Department of Pediatrics, Affiliated Hospital of Jiangnan University, Wuxi, Jiangsu, China; ^2^ Wuxi School of Medicine, Jiangnan University, Wuxi, Jiangsu, China

**Keywords:** Turner syndrome, cardiovascular abnormalities, children, warning signs, pregnancy hypertension

## Abstract

**Background/Objective:**

There are limited studies on cardiovascular abnormalities (CAs) and their warning signs in children with Turner syndrome (TS). The main aim of this 15-year retrospective study was to investigate the warning signs of CAs in children with TS and to suggest ways to prevent them.

**Methods:**

This retrospective study analyzed children diagnosed with TS at our pediatric endocrinology clinic. The study examined patients with TS with CAs and their warning signs.

**Results:**

A total of 37 cases were included in our study. The average age at presentation was 7.48 ± 3.49 years. According to the results of transthoracic echocardiography, the children were divided into two groups: (1) TS without CAs (*n* = 31) and (2) TS with CAs (*n* = 6). The incidence rate of CAs was 16.2% (6/37). Of the six cases, five had congenital heart disease, four of which underwent cardiac surgery. One patient developed descending aortic coarctation during growth hormone therapy and underwent aortic coarctation repair surgery. The proportions of haplotypes, chimeric types, and structural abnormalities in the TS without CAs group were 9/31, 16/31, and 6/31, respectively. In the TS with CAs group, these proportions were as follows: 1/6, 3/6, and 2/6, respectively. A comparative analysis revealed no statistically significant variation in karyotype frequencies between the two groups. In the TS without CAs group, 0.0% of the mothers had abnormal blood pressure during pregnancy. In the TS with CAs group, the incidence of hypertension in the mothers during pregnancy was 33.3%. A significant difference in gestational hypertension was observed between the two groups (*P* = 0.016). However, no significant differences were observed between the groups in terms of heart rate or blood pressure parameters (systolic/diastolic).

**Conclusions:**

Children with TS born to mothers with gestational hypertension appear to have a higher prevalence of CAs. These findings suggest that maternal gestational hypertension may serve as a potential early clinical marker for increased cardiovascular risk in this population and may warrant closer postnatal cardiac surveillance.

## 1 Introduction

Turner syndrome (TS) is the only monosomic syndrome that can be survived in humans. It has a prevalence of approximately 1 in 2,500 live-born female infants and 1 in 4,000 live-born infants (1). Cardiovascular abnormalities (CAs) occur in 50% of the TS population, which is a significant cause of premature death. This standardized mortality rate is three times higher than that in the general female population (2). In recent years, there has been an increase in studies on the TS population with CAs. TS may lead to the development of CAs at different stages of life. It is crucial to focus on the childhood phases of TS to improve the quality of life of these patients. Nevertheless, few studies have examined the rate of TS with CAs in children, nor have they investigated the associated warning signs.

Our study involved a retrospective analysis of children with TS with CAs, investigating the warning signs and providing ideas for relevant prevention and treatment measures. Children diagnosed with TS at our pediatric endocrine clinic and undergoing follow-up at outpatient clinics were included in our study.

## 2 Materials and methods

### 2.1 Patients

This was a retrospective, record-based study. Children who visited our pediatric endocrinology clinic with a diagnosis of “short stature, postnatal edema, no breast development, vaginal bleeding, and hypothyroidism” between January 2007 and December 2021 were included in the analysis. They were diagnosed with TS using a peripheral blood chromosomal karyotype. The study was approved by our medical ethics committee.

Enrollment criteria included complete personal and basic information, female external genitalia, and peripheral blood chromosome karyotype analysis meeting the following criteria: monosomic TS (45, X), chimeric TS (45,X/46,XX, 45, X/46,X,i(X), 45,X/47,XXX, 45,X[35]/46,X, r(X), etc.), and X chromosome structural abnormalities {partial deletions of the short arm of chromosome X, isochromosome Xq, ring X chromosome [r(X)], etc.} (3); they had transthoracic echocardiography reports.

Patients were excluded if they had (1) non-Turner syndrome karyotypes, (2) severely incomplete medical records, or (3) lacked transthoracic echocardiography reports.

### 2.2 Methods

The following clinical data were collected: birth date, birth weight, chronological age at presentation, height, weight, presence of Turner's signs, bone age (TW3 method), blood pressure (systolic blood pressure and diastolic blood pressure), heart rate, electrocardiogram (ECG), transthoracic echocardiography, history of cardiac surgery, other complications, whether or not the mother has gestational hypertension, and parental height. Body surface area was calculated in m^2^ as follows: <30 kg: weight × 0.035 + 0.1; >30 kg: (weight − 30) × 0.02 + 1.1. Body mass index (BMI) was calculated as follows (kg/m^2^): weight/(height × height). The BMI standard deviation score (SDS), height-for-age SDS, and height-for-bone-age SDS were calculated (4). Genetic target height was calculated in cm as follows: [(Paternal height + Maternal height) − 13 cm]/2.

Transthoracic echocardiography examinations at our hospital were performed by a team of three experienced ultrasound medicine specialists, each of whom has received specialized training in pediatric cardiology and echocardiography. The team adhered to standardized image acquisition and interpretation protocols to ensure consistent and reliable results.

Senior pediatric cardiologists with over a decade of clinical diagnostic experience confirmed the CA diagnoses, strictly adhering to established clinical guidelines. These specialists based their evaluations on thorough clinical examinations and transthoracic echocardiography.

The clinical parameters for a diagnosis of a CA were as follows: blood pressure ≥140/90 mmHg after 20 weeks of gestation, a negative urine protein test result or 24-h urine protein quantification of <0.3 g/L, with no history of hypertension or kidney disease before 20 weeks of gestation. The children were diagnosed with hypertension by referring to the Chinese blood pressure standard for those aged 3–17 years (5).

**Figure 1:**
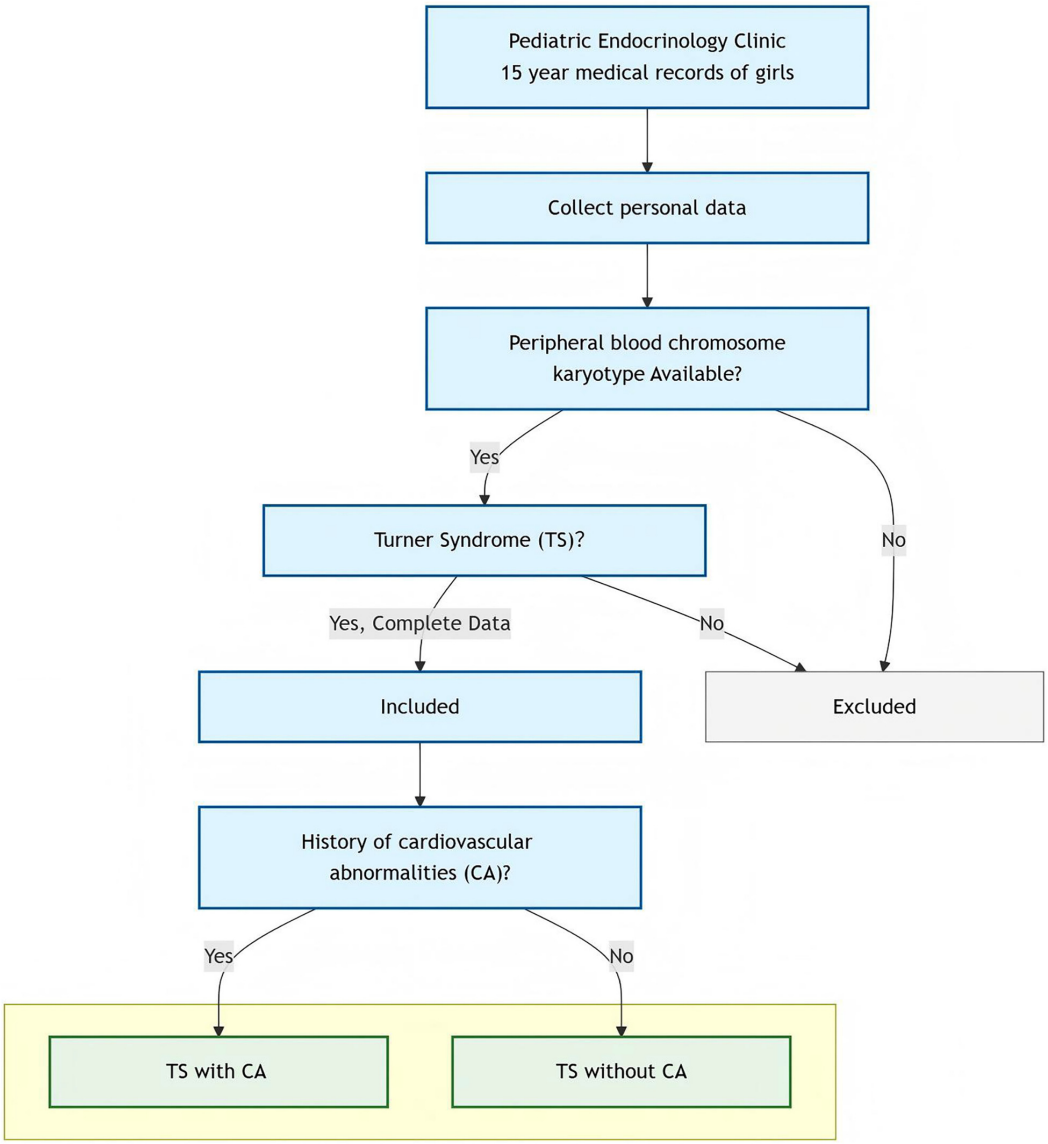
Patient screening flowchart.

Figure 1 shows the patient screening flowchart in detail.

### 2.3 Statistical analysis

The statistical analysis was performed using SPSS version 25.0. Measurement data were expressed as mean ± standard deviation. An independent samples *t*-test was used to compare the two groups for parametric data, and the Mann–Whitney *U*-test was used for non-parametric data. Enumeration data were expressed as the number of cases and percentage and analyzed using the chi-square (χ*^2^*) test or Fisher's exact probability method. A *P-*value of <0.05 was considered statistically significant. All figures were drawn using GraphPad Prism 9.5.0.

## 3 Results

### 3.1 Clinical characteristics and grouping of children with TS

A total of 47 cases of TS were successfully screened. Ten cases were excluded due to incomplete data and not meeting the inclusion criteria. Ultimately, 37 cases were included in the statistical analysis. Of the 37 cases, 32 had short stature, 1 had postnatal edema, 1 had a lack of breast development, 1 had vaginal bleeding, 1 had hypothyroidism, and 1 had delayed breast development. The mean birth weight was 2.87 ± 0.46 kg (range 2.00–3.50 kg). The mean age at presentation was 7.48 ± 3.49 years (range 1.08–15.63 years). The mean bone age was 6.54 ± 2.92 years (range 2.00–13.50 years). The mean height was 108.25 ± 16.95 cm (range 69.50–147.50 cm), and the mean weight was 20.07 ± 8.65 kg (range 7.94–50.00 kg).

**Figure 2:**
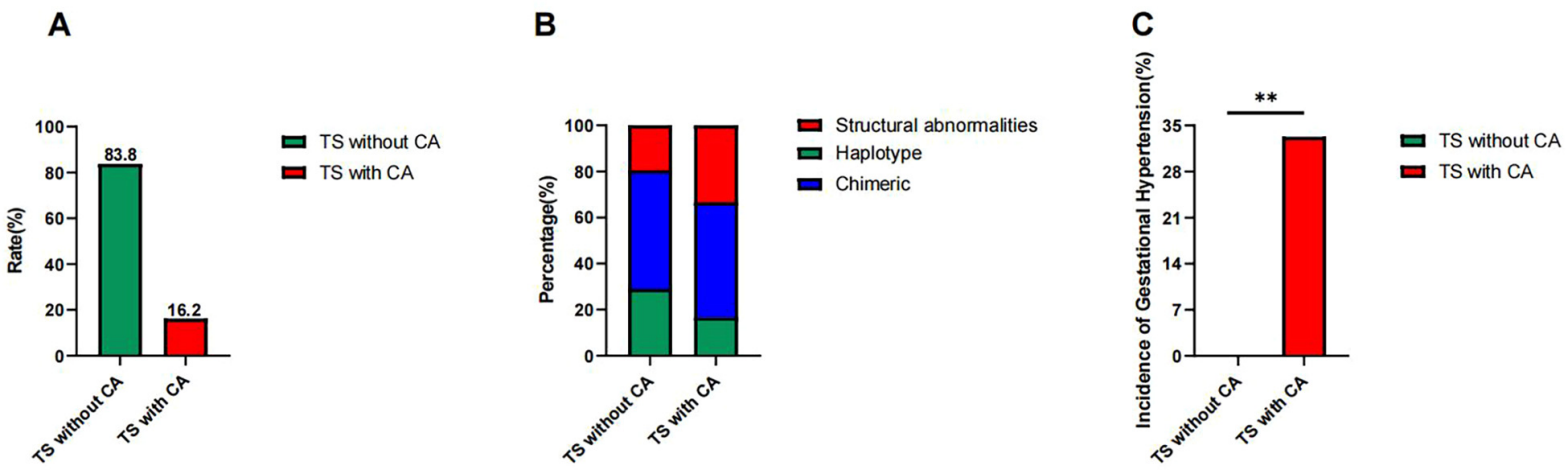
**(A)** Prevalence of CAs in children with Turner syndrome. The CA prevalence was 16.2% among the 37 children with TS, while the group without CAs accounted for 83.8%. **(B)** Distribution of haplotype, chimeric type, and structural abnormalities in children with Turner syndrome. The prevalence of haplotype, chimeric type, and structural abnormalities was 29.0%, 51.6%, and 19.4% in the TS without CAs group, respectively, while the prevalence in the TS with CAs group was 1/6 (16.7%), 3/6 (50.0%), and 2/6 (33.1%), respectively. **(C)** Incidence of gestational hypertension in the TS without the CAs and the TS with CAs groups. The incidence was significantly higher (33.3%) in the TS with CAs group compared to 0.0% in the TS without CAs group (**P = 0.016).

The 37 cases of TS were divided into two groups according to the presence or absence of CA: (1) TS without CAs (*n* = 31) and (2) TS with CAs (*n* = 6, 16.2%) (Figure 2A). There were no statistically significant differences in the following variables between the two groups: birth weight (kg), age at presentation (years), height (cm), weight (kg), BMI (kg/m^2^), body surface area (m^2^), bone age (TW3) (years), height-for-age standard deviation score, height-for-bone-age standard deviation score, heart rate (beats per min), systolic blood pressure (mmHg), diastolic blood pressure (mmHg), and genetic target height (cm). The parameters and corresponding *P-*values are shown in Table 1.

**Table 1:** Comparison of baseline characteristics between children with Turner syndrome with or without cardiovascular abnormalities.

Parameter	TS without the CAs group (*n* = 31)	TS with CAs group (*n* = 6)	*t*/*Z*-value	*P-*value
BW (kg)	2.86 ± 0.42	2.92 ± 0.65	0.293	0.771
CHA (years)	7.11 ± 3.22	9.39 ± 4.50	1.494	0.144
Height (cm)	106.50 ± 15.41	117.28 ± 22.98	1.448	0.157
Weight (kg)	19.17 ± 7.26	24.77 ± 13.81	1.476	0.149
BMI (kg/m^2^)	16.30 ± 2.02	16.76 ± 3.11	−0.124	0.902
BMI SDS	0.22 ± 1.12	−0.19 ± 0.76	−0.683	0.495
BSA (m^2^)	0.74 ± 0.18	0.88 ± 0.31	1.433	0.161
BA (TW3) (years)	6.17 ± 2.67	8.43 ± 3.63	1.791	0.082
AHSDS	−2.90 ± 0.50	−2.65 ± 0.59	1.083	0.286
BAHSDS	−2.21 ± 1.42	−2.28 ± 1.25	−0.097	0.924
HR (t/m)	96 ± 12	90 ± 11	−1.156	0.256
SBP (mmHg)	99 ± 6	104 ± 15	1.235	0.226
DBP (mmHg)	62 ± 6	67 ± 15	−0.413	0.679
GTH (cm)	157.97 ± 5.01	158.33 ± 2.46	0.166	0.869

TS, Turner syndrome; CAs, cardiovascular abnormalities; BW, birth weight, one data point of birth weight missing; CHA, chronological age; BMI, body mass index; BMISDS, body mass index standard deviation score; BSA, body surface area; BA, bone age; AHSDS, height-for-age standard deviation score; BAHSDS, height-for-bone-age standard deviation score; HR, heart rate; SBP, systolic blood pressure, four data points missing; DBP, diastolic blood pressure, four data points missing; GTH, genetic target height.

### 3.2 Differences in haplotypes, chimeric types, and structural abnormalities between the TS without CAs and the TS with CAs groups

The frequency of haplotypes, chimeric types, and structural abnormalities was 9/31 (29.0%), 16/31 (51.6%), and 6/31 (19.4%) in the TS without CAs group, respectively, and 1/6 (16.7%), 3/6 (50.0%), and 2/6 (33.1%) in the TS with CAs group, respectively. There was no statistical difference in the proportion of haplotypes (χ*^2^* = 0.390, *P* = 0.532), mosaic type (χ*^2^* = 0.005, *P* = 0.942), or structural abnormality (χ*^2^* = 0.580, *P* = 0.446) between the two groups. These details are shown in Table 2 and Figure 2B.

**Table 2:** Peripheral blood karyotypes in 37 children with Turner syndrome.

Group	No.	Karyotype
(1) TS without CAs	1	45,X[50]
	2	46,X,i(X)(q10)
	3	45,X[58]/46,x,+mar[30]/46,X,r(X)(p22.3q28)[12]
	4	45,X[39]/46,X,i(X)(q10)[11]
	5	45,X[16]/46,X,i(X)(q10)[44]
	6	45,X[54]/46,X,i(X)(q10)[6]
	7	45,X[21]/46,X,i(Xq)[9]
	8	45,X[7]/46,X,i(X)(q10)[43]
	9	45,X
	10	45,X
	11	45,X[30]/46,X,psu idic(X)(q22.1)[30]
	12	46,X,i(X)(q10)[30]
	13	45,X[13]/46,X,i(X)(q10)[47]
	14	45,X[47]/47,XXX[12]
	15	45,X[51]/47,XXX[9]
	16	45,X[45]/47,XXX[15]
	17	46,X,i(X)(q10)
	18	45,X[35]/46,X, r(X)(p22.3q28)X[15]
	19	46,X,i(X)(q10)
	20	45,X[9]/46,X,i(X)(q10)[41]
	21	45,X[5]/46,X,i(X)(q10)[37]/46,XX[8]
	22	45,X[37]/46,XX[13]
	23	45,X[52]/47,XXX[8]
	24	45,X[50]
	25	45,X[33]/46,X,i(X)(q10)[27]
	26	45,X
	27	45,X
	28	45,X
	29	45,X
	30	45,X
	31	46,X,del(X)(p21.2),inv(9)(p12q13)
(2) TS with CAs	32	46,X,del(X)(p10)
	33	45,X[44]/46,X,i(X)(q10)[16]
	34	45,X[57]/46,X,+mar[3]
	35	46,X,i(X)(q10)
	36	45,X[30]
	37	45,X[6]/46,X,del(X)(p11.2)[54]

TS, Turner syndrome.

### 3.3 The difference in gestational hypertension between the TS without CAs and the TS with CAs groups

The incidence of gestational blood pressure abnormalities in the TS without CAs group was 0.0% (0/31). Two cases of gestational hypertension and one case of hypotension occurred in the TS with CAs group, with an incidence rate of gestational hypertension of 33.3% (2/6). Fisher's exact test revealed a statistically significant difference in the incidence of gestational hypertension between the two groups (*P* = 0.016) (Figure 2C). The incidence of gestational hypotension (16.7%, 1/6) was not statistically significant compared to the TS without CAs group (*P* = 0.114). This suggests that gestational hypertension may be a warning sign of TS with CAs, whereas gestational hypotension may not be.

Heart rate and blood pressure data were missing for 4 of the 31 cases in the TS without CAs group. The incidence of an increased heart rate in the TS without CAs group was 18.5% (5/27), while elevated systolic and diastolic blood pressure occurred in 3.7% (1/27) and 7.4% (2/27) of the cases, respectively. The incidence of an increased heart rate in the TS with CAs group was 0.0% (0/6). The incidence of increased systolic blood pressure and diastolic blood pressure was 16.7% (1/6), respectively. There were no statistically significant differences in the incidence of increased heart rate, systolic blood pressure, and diastolic blood pressure between the two groups (*P* = 0.556, 0.335, and 0.464, respectively). This suggests that children with TS have the same probability of increased heart rate, systolic blood pressure, and diastolic blood pressure, regardless of whether they have a CA. These indicators may not be statistically significant warning signs for TS with CAs.

### 3.4 ECG abnormalities in the TS without CAs and TS with CAs groups

ECG abnormalities were observed in seven cases in the TS without CAs group, and ECG data were missing in six cases, representing an incidence rate of 28.0% (7/25). Four patients had sinus tachycardia. Two patients had changes to their ST segment, and one patient had an incomplete right bundle branch block. There were three cases with ECG abnormalities in the TS with CAs group (3/6), with an incidence rate of 50.0%. Fisher's exact test revealed no statistical significance when comparing the incidence rates of the two groups (*P* = 0.358).

The incidence of being small for gestational age was 22.3% (7/30; data for birth weight were missing for 1 case) in the TS without CAs group and 33.3% (2/6) in the TS with CAs group. This difference was not statistically significant (*P* = 0.627).

### 3.5 Transthoracic echocardiography data of the TS with CAs group

A total of six cases in the TS with CAs group were diagnosed at a median age of 10.4 years (range 0.1–15.6 years). The cardiovascular abnormalities were found at a median age of 7.2 years (range 0.2–15.8 years). Of the six cases, five had congenital heart disease. Four of them underwent cardiac surgery (surgical rate of 66.7%). The five cases included a ventricular septal defect (subpulmonary type, approximately 8 mm) with the formation of a membranous aneurysm, spontaneous healing of a ventricular septal defect (5.5 mm), widening of the aortic root with mild left ventricular enlargement, a secundum atrial septal defect (16 mm), and a partial anomalous pulmonary venous connection with an atrial septal defect. One patient developed descending aortic coarctation during growth hormone therapy and underwent aortic coarctation repair surgery.

## 4 Discussion

Our study suggests that gestational hypertension may serve as a potential warning sign for TS with CAs in children. A study showed that infants born to women with pre-eclampsia were more likely to have congenital heart defects than those born to women without the condition (6). The prevalence of septal defects was highest among infants born to women with early-onset pre-eclampsia (before 34 weeks), compared to those with later onset (6). Of the six children in our study who had TS with CA, two had mothers with gestational hypertension. In particular, one infant was small for gestational age with a birth weight of 2.25 kg. This is consistent with studies reporting that arterial hypertension during pregnancy can increase the risk of low birth weight and infants who are small for gestational age (7). Gestational hypertension in mothers appears to be associated with CA and/or low birth weight in female infants, warranting consideration of TS in these infants. These findings suggest a possible association between gestational hypertension and CA in children with TS, though causality remains unconfirmed and requires further study.

Transthoracic echocardiography (or cardiovascular MRI) is the primary method for the diagnosis of CA and ongoing monitoring in children with TS (8). Transthoracic echocardiography is routinely recommended as the initial imaging modality upon TS diagnosis. There is a high risk of cardiovascular complications, particularly aortic dilation, in children with TS, and our study found a CA prevalence of 9%, within the reported literature range of 1%–45% (9). The most common CAs in TS include a bicuspid aortic valve (20%–30%) and coarctation of the aorta (7%–18%) (10). Our study identified two cases of aortic coarctation (5.4%, 2/37), suggesting a relatively low incidence. This discrepancy may reflect that CA prevalence in TS is typically reported across all age groups, whereas our study specifically focused on pediatric cases. Some cardiac abnormalities develop with age, and some children in this study have not yet reached the age of onset of these CAs. Diagnosis is also limited by transthoracic echocardiography technology. Therefore, it is essential to follow up on cardiovascular changes in children with TS closely.

Due to the high risk of cardiovascular complications, it is recommended to initiate cardiac-related examinations in patients with TS at 12 years (11). The mean age of aortic dilation onset was 25.55 ± 5.78 years (12), and the median age of onset of aortic dissection was 35 years (13). In our study, the median age of onset of cardiac abnormalities was 7.2 years (0.2–15.8 years). One of the 37 cases had aortic root widening at the age of 4.3 years, which was significantly younger than the onset age reported in the literature, suggesting that cardiovascular changes in children with TS younger than 12 years old should be monitored.

There were six children with TS with confirmed cardiac abnormalities in our study. Four of them underwent cardiac surgery, with an operation rate of 66.7%, among which surgery for coarctation of the aorta made up 50%. As reported in the literature (14), among 780 patients with TS aged 0–18 years who required surgery, 274 (35%) underwent repair of coarctation of the aorta and 116 (15%) underwent repair of the aortic arch. The proportion of children with TS receiving surgery for coarctation of the aorta in our study was higher than in the studies referenced above, which may be related to the small number of total cases with cardiac abnormalities in our study. This suggests that the possibility of coarctation of the aorta should be regularly monitored using transthoracic echocardiography in children with TS. At least 12.6% of girls born with aortic coarctation are diagnosed with TS (15), indicating that if these girls are diagnosed with aortic coarctation via transthoracic echocardiography during the fetal period or after birth, the possibility of TS should be considered. Peripheral blood karyotype analysis can aid in further diagnosis.

TS populations have exhibited significantly elevated systolic and diastolic blood pressure, heightening cardiovascular complication risks (16). In our study, although there was no statistical difference in the occurrence of increased blood pressure between the two groups, the rate of increased blood pressure was higher in the TS with CAs group. One study demonstrated that pediatric patients with TS and hypertension had heightened cardiovascular disease predisposition, attributable to metabolic dysregulation (17). Our study revealed that while neither systolic nor diastolic hypertension appeared to directly cause CAs in the children with TS, patients with existing cardiac anomalies require regular blood pressure surveillance for optimal complication prevention.

This study was limited by its retrospective design, small sample size, and incomplete imaging data, which restricted the ability to draw definitive conclusions. As a single-center cohort, the findings may not be broadly generalizable. In addition, the sample size was insufficient to fully assess all the statistical associations explored, including the relationship between gestational hypertension and CA in patients with TS. Nonetheless, this study raises important concerns and highlights patterns that warrant further investigation in larger, prospective multicenter cohorts using age-specific surveillance protocols.

## 5 Conclusion

Children with TS born to mothers with gestational hypertension appear to have a higher prevalence of CAs. These findings suggest that maternal gestational hypertension may serve as a potential early clinical marker for increased cardiovascular risk in this population and may warrant closer postnatal cardiac surveillance.

## Data Availability

The original contributions presented in the study are included in the article/Supplementary Material, further inquiries can be directed to the corresponding authors.
